# Scattering-angle-dependent Christiansen color spectra data of poly(vinyl chloride) (PVC) suspended in styrene liquid and a comprehensive data list of wavelength-dependent refractive indices of PVC

**DOI:** 10.1016/j.dib.2018.08.101

**Published:** 2018-08-30

**Authors:** Sadaki Samitsu, Hideki T. Miyazaki, Hiroyo Segawa

**Affiliations:** a Data-driven Polymer Design Group, Research and Services Division of Materials Data and Integrated System (MaDIS), National Institute for Materials Science (NIMS), 1-2-1 Sengen, Tsukuba, Ibaraki 305-0047, Japan; b Research Center for Functional Materials, NIMS, 1-1, Namiki, Tsukuba, Ibaraki 305-0044, Japan

## Abstract

This paper reports transmission and scattering spectra of poly(vinyl chloride) (PVC) in styrene liquid, which is derived from Christiansen effect. The spectra were measured by varying scattering angles. Further discussion on Christiansen color was provided in the paper entitled "Transmitting and scattering colors of porous particles of poly(vinyl chloride) based on Christiansen effect" (Samitsu et al., 2018) [1]. The paper additionally provides refractive indices of PVC reported in literatures because Christiansen effect has close relationship with wavelength-dependent refractive index, i.e. optical dispersion. The values have considerable range probably depending on samples and determination methods for refractive index. The comprehensive data list is therefore potentially useful for studying refractive index of polymers.

## Specifications table

**Table:** 

Subject area	*Chemistry*
More specific subject area	*Optical properties of polymers*
Type of data	*Table, graph, text file*
How data was acquired	*Optical measurement, Numerical calculation*
Data format	*Analyzed*
Experimental factors	*Poly(vinyl chloride) (TH-500 Taiyo Vinyl Corporation)powder is immersed into styrene liquid.*
Experimental features	*Scattering angle dependent scattering spectra experimentally recorded and numerically calculated.*
Data source location	*National Institute for Materials Science, Japan.*
Data accessibility	*Data is available with the article.*

## Value of the data

• Scattering spectra of the Christiansen coloration of PVC are provided with respect to various scattering angles.
• Single scattering efficiency of a PVC sphere located in styrene liquid is presented, which was numerically calculated based on the rigorous Mie scattering theory.
• Refractive index values of PVC reported in handbooks and literatures are summarized.

## 1 Data

**Fig. 1:**
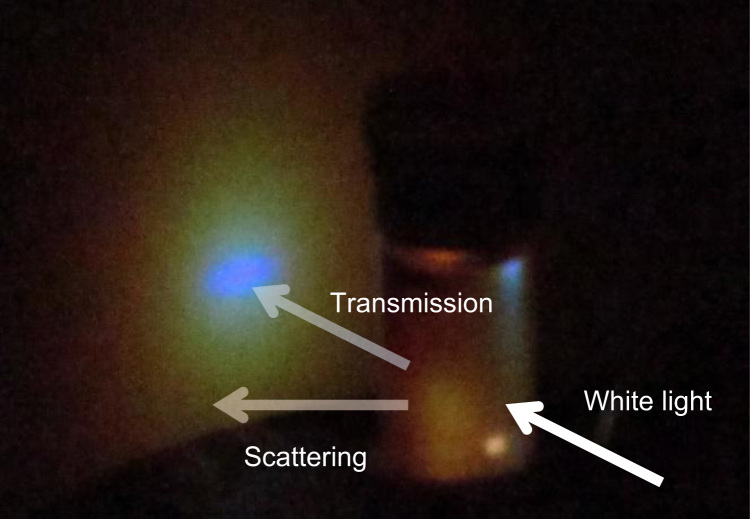
A photographic image of a PVC suspension irradiated by white light.

**Fig. 2:**
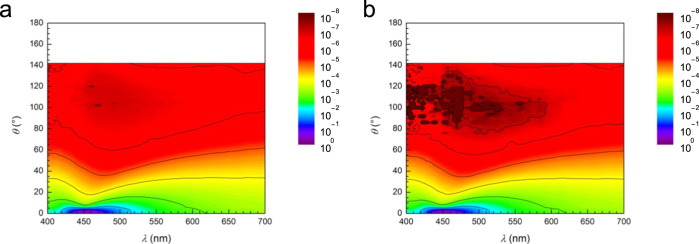
Experimentally recorded scattering spectra of a PVC suspension plotted as a function of scattering angle for V-polarized (a) and H-polarized incidence (b). The scattering angle *θ* = 0° corresponds to the transmission spectrum.

**Fig. 3:**
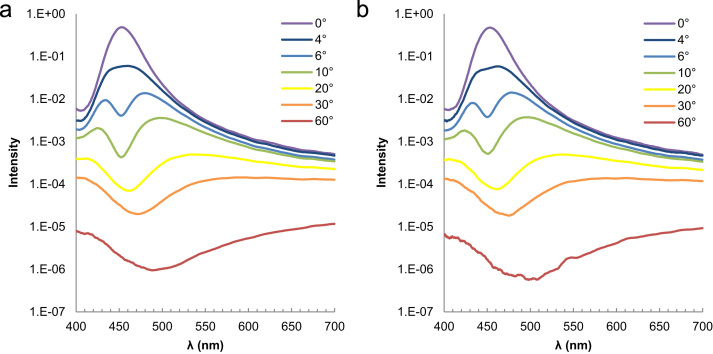
Scattering spectra of a PVC suspension at the typical scattering angles for V-polarized (a) and H-polarized incidence (b).

**Fig. 4:**
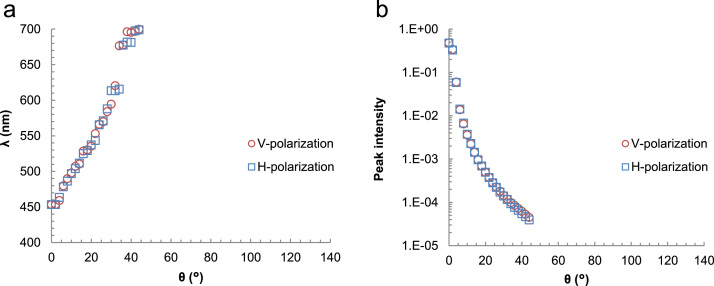
(a) Peak wavelength and (b) peak intensity of the scattering spectra shown in Fig. 2, plotted as a function of scattering angle. The peaks could not be determined at the *θ* ≥ 44° because the peak maximum moved out of 700 nm.

Fig. 1 shows a photographic image of a PVC suspension, and Fig. 2 displays the experimentally recorded scattering spectra plotted as a function of the scattering angle on the vertical axis. While uniform transmission color, independent of incident angles, was reported by Takeoka et al. [2], no study has been performed for the scattering angle dependence of the scattering spectra of Christiansen coloration. Both V- and H-polarized incidences resulted in similar scattering spectra, indicating that polarization does not have a large influence on the Christiansen effect. Fig. 3 shows the scattering spectra at the typical scattering angles. A transmission spectrum corresponding to *θ* = 0° exhibits a strong peak at 455 nm, which agrees with the transmission spectrum shown in Fig. 3 of the paper [1]. The scattering spectra recorded at low *θ,* ≤ 4°, included transmitted light to some extent, which displayed a relative intensity near 455 nm. This beam divergence is because (1) the incident light is refracted at the surfaces of the cylindrical vial by 3°, and (2) weakly scattered light with a wavelength near that of the transmission peak slightly changed its direction while passing through a thick suspension. As the scattering angles increased in the narrow *θ* range 4–30°, the peaks of the scattering spectra shifted to longer wavelengths, as shown in Fig. 4(a), which continuously changed the colors of the scattered light: blue, green, yellow, and orange from low to large *θ*. At large *θ* ≥ 30°, the scattered light includes a wide wavelength range that is exclusive of the dip at 455 nm. The scattered light exhibits red color, independent of the scattering angles at large *θ* ≥ 30°. Peak wavelengths could not be determined at the *θ* ≥ 44° because the peak maximum moved out of 700 nm, the maximum wavelength available in this experiment. An increase in the scattering angle significantly decreases the scattering intensity by a few orders of magnitude (Fig. 4(b)).

**Fig. 5:**
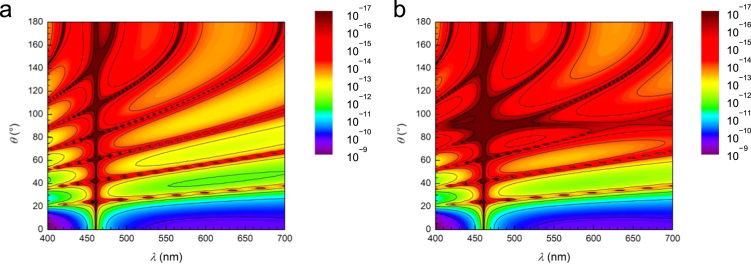
Single scattering efficiency of a PVC sphere located in styrene liquid at different scattering angles. The efficiency was calculated for V-polarization (a) and H-polarization (b) of the incident light based on the rigorous Mie scattering theory.

Fig. 5 shows the single scattering efficiency of a PVC sphere in styrene. Both V- and H-polarized incidences show similar scattering efficiency due to the symmetry of the sphere. Based on the result, polarization of light was not considered in the main text of the paper [1]. The calculated spectra exhibit many fringes, resulting from resonant Mie scattering. The fringe position, i.e., the wavelength of dip, varies markedly with the scattering angle. However, such fringes, a feature of wave optics, were blurred out in the experimental spectra shown in Fig. 2 because these spectra arise from the multiple scatterings of irregular particles having different sizes and shapes. This indicates that the uniform scattering color at large *θ,* ≥ 30°, originates from the multiple scatterings of irregular particles, and is not a feature of single scattering by a definite-shaped particle. The single scattering efficiency of a particle is significantly small: for example, the scattering intensity of light at *λ* = 600 nm, *θ* = 10°, and *r* = 25 mm is calculated to be of the order of 10^−10^. On the other hand, the number of particles existing in the sample volume corresponding to the optical path (1.2 mm in diameter and 18 mm in length) is roughly estimated as 10^10^. An enormous number of multiple scatterings in a thick sample can enhance the weak intensity of the single-scattered light so strongly that a vivid scattering color can be observed. Owing to the weak scattering intensity, the Christiansen coloration detectable by the naked eye requires a sample thickness at least of the order of millimeters. The calculation does not agree completely with the experiments due to the undefined factors in the experimental data: (1) number of multiple scattering events, (2) size distribution, and (3) irregular shapes of PVC particles. In spite of the disagreement, the numerical calculation of the single-scattering efficiency at different scattering angles reproduces the overall feature of the observed Christiansen effect; this effect results from an ensemble of multiple scattering events involving irregular-shaped particles having uniform refractive indices. Refractive indices of PVC reported in papers and handbooks were summarized in Table 1.

**Table 1:** List of refractive indices of PVC reported in literature.

***λ* (nm)**	***n***_**589**_	***T*****(°C)**	**Ref.**	**Remark**
486.1	1.54806		[3]	
589.3	1.54151		[3]	
656.3	1.53843		[3]	
589.3	1.54–1.55		[4]	
589.3	1.52–1.57		[5]	
589	1.4831	20	[5]	
589	1.5381	20	[5]	
589	1.5066	20	[5]	PVC molding compound—rigid
589	1.541	25	[5]	
589	1.484	25	[5]	rigid
589	1.52–1.55		[5]	rigid
589	1.539		[6]	
589	1.539		[7]	
–	1.548	27	[8]	Method: grain immersion method
589	1.55		[9]	
589	1.57		[10]	Method: Benford refractometer
589	1.544		[7]	Calculated from R_LL_^a^
589	1.543		[7]	Calculated from R_GD_^b^
589	1.511		[7]	Calculated from R_V_^c^

a $n=\;{\left(\frac{1+2\frac{R_{\mathrm{LL}}}{V}}{1-\frac{R_{\mathrm{LL}}}{V}}\right)}^{\frac{1}{2}}$.

b $n=1+\frac{R_{\mathrm{GD}}}{V}$.

c $n=\frac{R_{V}}{M}$.

## 2 Experimental design, materials and methods

### 2.1 Experimental setup for scattering angle dependence of Christiansen effect

**Fig. 6:**
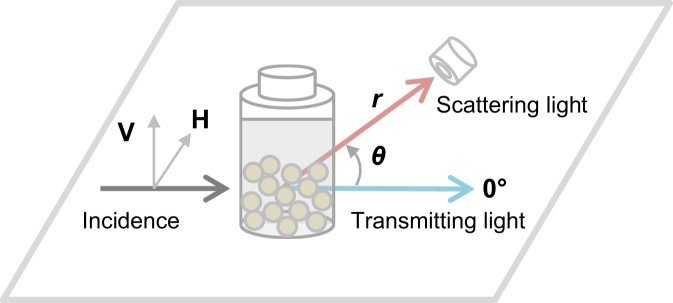
An optical arrangement of the measurement setup. A fiber probe connected to a spectrometer with an effective opening size of 2 mm was rotated around a sample vial; the probe was positioned *r* = 25 mm from the center of the vial. The available *θ* for measuring the scattering spectrum was in the range 0–142° for this setup.

A collimated beam of white light was incident on a PVC suspension and its scattering spectra were recorded while changing the scattering angle *θ*. Fig. 6 illustrates an optical arrangement of the measurement setup. The beam, with a diameter of 1.2 mm, was passed through a polarizer and subsequently irradiated to a cylindrical-shaped glass vial with a diameter of 18 mm. A fiber probe connected to a liquid nitrogen-cooled CCD spectrometer was rotated around the sample vial, while maintaining a distance *r* = 25 mm from the center of the vial. The effective opening size of the probe was 2 mm. The scattering spectra were recorded every 2° for *θ* = 0–142°, and each spectrum was normalized to the spectrum of incident light. The effective wavelength of the spectra ranged 400–700 nm, depending on the specifications of the light source, lens, polarizer, sample vial, and detector. The electric fields of polarized light perpendicular and parallel to the rotation plane are denoted as V- and H-polarization, respectively. Regarding the V- and H-polarizations of the incident beam, the scattering spectra were recorded separately.

### 2.2 Numerical calculation procedure of scattering angle dependence of Christiansen effect

The angle-dependent single-scattering efficiency of a sphere for every 2° of *θ* (0–180°) were numerically calculated based on the rigorous Mie scattering theory for V- and H-polarized incidence. We considered a PVC sphere with a diameter of 1 μm that is placed in styrene liquid. Details of numerical calculation is described in [1]. For PVC particle and styrene, the *n*(*λ*) plotted in Fig. 6 of the paper [1] are used. The single-scattering efficiencies are given as *S*_V_*λ*^2^/(4π^2^*r*^2^) and *S*_H_*λ*^2^/(4π^2^*r*^2^) for V- and H-polarized incidences, respectively, where *S*_V_ and *S*_H_ are the scattered irradiance per unit incident irradiance for the respective polarizations of the incident beams [11].
