# Supplementary material for: Scattering-angle-dependent Christiansen color spectra data of poly(vinyl chloride) (PVC) suspended in styrene liquid and a comprehensive data list of wavelength-dependent refractive indices of PVC
Source: Data Brief. 2018 Aug 30;20:1099–104. doi: 10.1016/j.dib.2018.08.101 (PMC6139890; doi:10.1016/j.dib.2018.08.101)
Supplement: Supplementary file 1 — Supplementary material [file mmc1.pdf]

NATIONAL INSTITUTE FOR MATERIALS SCIENCE
1-2-1, SENGEN, TSUKUBA, IBARAKI 305-0047, JAPAN

PHONE: +81-29-860-4745
FAX: +81-29-859-2121
E-mail: SAMITSU.Sadaki@nims.go.jp

July 10th, 2018
Dr. Sadaki Samitsu
Senior Researcher

Dear Managing Editor in *Data in Brief*,

We declare herein that our revised manuscript (DIB-D-18-01424) entitled "Scattering-angle-dependent Christiansen color spectra data of poly(vinyl chloride) (PVC) suspended in styrene liquid and a comprehensive data list of wavelength-dependent refractive indices of PVC," is original, has not been previously published in whole or in part, and is not under consideration for publication in any other journal. The research has been conducted by receiving financial support from JSPS KAKENHI Grant Number JP 26410230 and 17K06007, which was described in Acknowledgement section of the paper. We declare no competing financial interest in this study. All authors agreed with submission of the paper to *Data in Brief*.

I would like to thank you in advance for considering this manuscript. Please feel free to contact me if you have any questions or require further information.

Sincerely,

Sadaki Samitsu

Dr. Sadaki Samitsu,
Senior Researcher, Data-driven Polymer Design Group
Research and Services Division of Materials Data and Integrated System (MaDIS),
National Institute for Materials Science (NIMS)
1-2-1 Sengen, Tsukuba, Ibaraki 305-0047, Japan
TEL: +81-29-860-4745, FAX: +81-29-859-2121
E-mail: SAMITSU.Sadaki@nims.go.jp
